# Effect of Dietary Ethanolic Extract of *Lavandula officinalis* on Serum Lipids Profile in Rats

**Published:** 2014

**Authors:** Zahra Rabiei, Mahmoud Rafieian-Kopaei, Shiva Mokhtari, Mehrdad Shahrani

**Affiliations:** *Medical Plants Research Center, Shahrekord University of Medical Sciences, Shahrekord, Iran.*

**Keywords:** *Lavandula officinalis*, Serum lipids, Atherogenic index, Cardiovascular

## Abstract

Antioxidants are effective in prevention and treatment of cardiovascular diseases. *Lavandula officinalis *possesses antioxidant activity, therefore, in this study; the effects of *Lavandula officinalis* extract were investigated on serum lipids levels of rats. Experimental mature male Wistar rats were treated with 100, 200 or 400 mg/Kg/day of lavender ethanolic extract or distilled water for 25 days via gastric gavage (n=8 each group). At the end of 25^th^ day, the serum cholesterol, triglyceride, HDL, LDL and VLDL levels, as well as atherogenic indices were determined in rats’ serum. The ethanolic extract of lavender decreased serum cholesterol, triglyceride, LDL and VLDL levels in 100 mg/Kg group (p=0.03, p=0.001, p=0.001, p=0.001, respectively). Serum HDL level increased in 100 mg/Kg/day group (p=0.01). Lavender extract decreased LDL/HDL level at doses of 100 and 200 mg/Kg/day (p=0.001, p=0.001, respectively). The TG/HDL levels decreased in experimental groups with doses of 100 and 200 mg/Kg/day (p=0.001, p=0.001, respectively). *Lavandula officinalis *extract exerts hypolipidemic effect in rats and might be beneficial in hyperlipidemic patients.

## Introduction

Cardiovascular diseases are the leading causes of death in the United States of America. High levels of plasma cholesterol, especially the LDL (low density lipoprotein) is known as important risk factors for coronary heart disease (CHD). Whereas high levels of HDL reduces the risk factors of CHD (1). It is now generally accepted that oxidation of low density lipoprotein (LDL) plays a significant pathogenic role in atherosclerosis (2). Atherosclerosis is a chronic disease of large and medium sized arteries with hardening and loss of elasticity of the arterial walls and narrowing of the arterial lumen. Atherosclerosis is the principal cause of cardiovascular and cerebrovascular diseases leading to angina pectoris, myocardial infarction and ischemic stroke and is the principal cause of death in Western countries (5). Fatty acid composition of dietary influences on blood lipids and blood lipoproteins are associated with the development of atherosclerosis and ischemic heart disease (1). The process of atherogenesis has been considered by many to consist largely of the accumulation of lipids within the artery wall. High plasma concentrations of lipids, in particular those of low-density lipoprotein (LDL) cholesterol, are the principal risk factors for atherosclerosis (6). There are several groups of drugs for hyperlipidemia, however, most of them possess high adverse effects (7). Nowadays, there has been a great increase in the use of complementary treatments such as herbal remedies in the treatment of diseases (8). 

*Lavandula officinalis* (family: Lamiaceae, Synonyms: *Lavandula officinalis* Chaix *ex *Vill), commonly known in Iran as “stoechas”, is one of these plants which has been traditionally used for different diseases (9). Phytochemical studies revealed that linalool, linalyl acetate and some other monoterpene and sesquiterpenes, flavonoids like luteolin, triterpenoids like ursolic acid and coumarins like umbelliferone and coumarin were the main components of the aerial parts and flowers of *Lavandula officinalis* which might be effective on serum lipids levels (10). Following oral administration of this plant extract at the doses of 500, 1000, 1500, 2000, 3000, and 5000 mg/Kg no toxicity and no significant changes in the body weight have been reported in control and treated groups (11). Treatment with Lavender oil significantly decreased neurological deficit scores, infarct size, the levels of Malondialdehyde (MDA), carbonyl and reactive oxygen species (ROS), and attenuated neuronal damage, upregulated superoxide dismutase (SOD), catalase (CAT), GSH-Px activities and GSH/GSSG ratio. It has also shown to have neuroprotective activity against cerebral ischemia/reperfusion injury which might be attributed to its antioxidant effects (12). This plant possesses high levels of polyphenol compounds having antioxidant properties. Antioxidants are effective in prevention and treatment of cardiovascular diseases, particularly, atherosclerosis (13).

Therefore, this study was aimed to determine the composition of polyphenols, phenols and antioxidant properties of Lavender extract, other than evaluating its effect on blood lipids profile.

## Experimental

*Plant material and preparation of extracts*

The plant was purchased from a local provider in Shahrekord, and authenticated in Medical Plants Research Center, Shahrekord University of Medical Sciences. A voucher specimen was deposited in Herbarium unit of this university (No. 421). For preparation of ethanolic extract, air-dried and powdered flowering branches of the plant were macerated with ethanol (96%, Ghadir Industries, Iran) for 48 h. The macerated powder was then shacked, filtered and evaporated in a rotary evaporator under reduced pressure until dryness (15)**.**

*Total phenolic compounds determination*

The amount of total phenolic compounds in the *Lavandula officinalis* extract was determined colorimetrically with Folin-Ciocalteu reagent. Mixed with the Folin-Ciocalteu reagent (1:10 diluted with distilled water) and aqueous Na2CO3 (0.4 mL, 7.5%) was added 0.5 mL of the extract. The mixture was allowed to stand for 30 min and the total phenols were determined by spectrophotometer (Unico UV-2100, USA) at 765 nm. A standard curve was prepared using 12.5, 25, 50, 62.5, 100 and 125 mg/L solutions of gallic acid in methanol and water (60:40, v/v). Total phenol values were expressed in terms of gallic acid equivalent (mg/g), which is a common reference compound. The experiment was repeated 3 times (16). 

*Total flavonoid and flavonol determination*

The amount of total flavonoids in the *Lavandula officinalis* extract was determined using the colorimetric method. Mixed with 1.5 mL of methanol (60%), 1 mL of 2% aluminum chloride, and 6 mL of 5% potassium acetate was added 1 mL of the Lavender extract or rutin. The mixture was left at room temperature for 40 min. The absorbance of the reaction mixture was then measured at 415 nm. The calibration curve was prepared using rutin solutions at concentrations of 25, 50, 100, 250 and 500 ppm in methanol. The aluminum chloride colorimetric method was employed for flavonol determination, but the incubation period was 150 min and the absorbance of the reaction mixture was determined at 440 nm. The experiments were repeated 3 times. Total flavonoids and flavonols were expressed in terms of rutin equivalent (mg/g), which is a common reference compound (17). 

*Measurement of the antioxidant activity by Beta-carotene- linoleate method*

Beta-carotene-linoleate method was used for measuring antioxidant capacity. Two milligrams of betacarotene was solved in 2.0 mL of chloroform. 20 mg linoleic acid and 200 mg tween 40 were added to the emulsion. 40 mL of water saturated with oxygen was added to the above materials. *Lavandula officinalis* extract was prepared at a concentration of 2.0 milligrams per liter in pure ethanol. 4 mL of the prepared solution was added to 2 mL of the prepared extract and control (ethanol). Antioxidant activity of the extract based on Beta-carotene bleaching rate at a wavelength of 470 nm for a period of 180 minutes (15 min intervals) was calculated using the following formula. The mean of obtained values was considered as the percent of antioxidant activity of the extract (18). 

AA = 100 × [1 - (A_0_-A_t_) / (A0_0_ – A0t)]

AA: antioxidant activity

A_0_ : sample absorbance at time zero 

A_t _: control absorbance at different times during 180 minutes 

A0_0_ : sample absorbance at time zero 

A0_t _: control absorbance at different times during 180 minutes 

*Animals*

All experimental animal procedures were conducted with the approval of the Ethics Committee of Shahrekord University of Medical Science, Iran. Male Wister rats (body weight 200-250 g) were housed under conditions of controlled temperature (22 ± 2 ^◦^C) and constant humidity, with 12 h light/dark cycle (light on 07:00-19:00). Food and water were available ad libitum. The rats were divided into 4 groups (8 animals per group). Control group received gastric gavage of distilled water and three treatment groups received Lavender ethanolic extract with doses of 100, 200, 400 mg/Kg/day for 25 days. On the 25^th^ day of the experiment the blood lipid profile was determined. Doses were chosen based on previous studies (11).

*Lipid profiles*

At the end of 25^th^ day, all animals were anesthetized with chloral hydrate (400 mg/Kg *i.p*.) and a venous blood sample was drawn and centrifuged at 7000 g for 10 min. The supernatant (serum) was frozen at −20 ^◦^C until assay for HDL, LDL, VLDL, triglyceride, and cholesterol content (Pars Azmun, Iran according to manufacturer's instructions) using auto-analyzer (Liasys, Roma, Italy) (19).

*Statistical analysis*

Cholesterol, triglyceride, LDL, HDL, and VLDL were analyzed using one-way ANOVA test (post hoc LSD). Data were expressed as means ± SEM. P < 0.05 was considered significant.

## Results

*Analysis of Lavandula officinalis extract*

The amount of phenolic compounds was 79.1 mg/g of dry extract equivalent to gallic acid. The amounts of flavonol and flavonoid compounds were 45 mg/g and 21.8 mg/g dry extract, equivalent to Rutin. The antioxidant activity of Lavender extract was 40% as compared to Beta-caroten.

*Effects of ethanolic extract of Lavandula officinalis on serum lipids profile*

**Figure 1:**
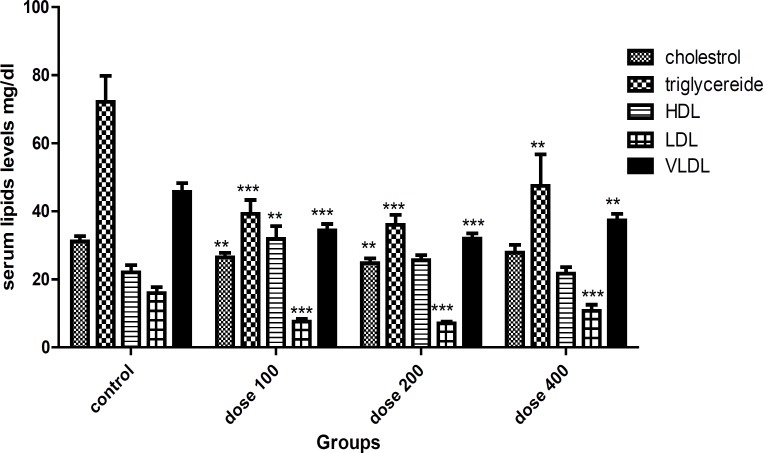
Serum lipids levels (mg/dl) (**=P < 0.05; ***=P < 0.01) n=8

**Figure 2:**
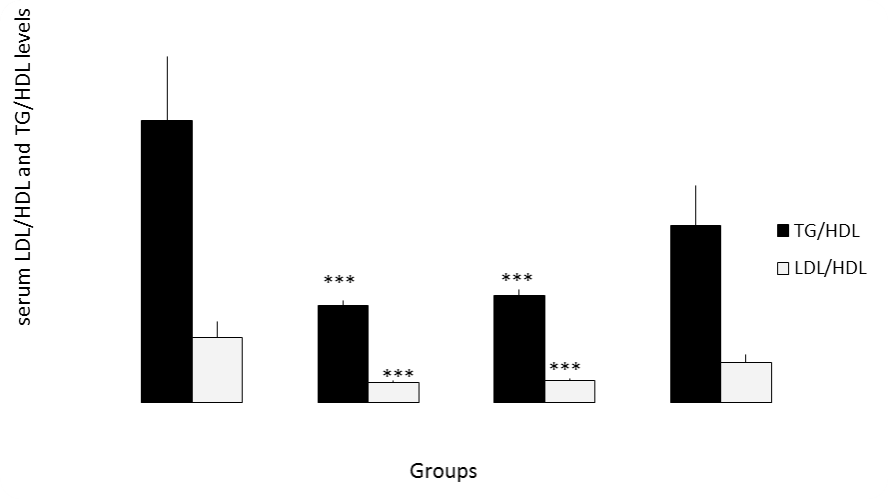
Serum LDL/HDL and TG/HDL levels. (***=P < 0.01) (n=8).

Serum cholesterol level was lower in the 100 and 200 mg/Kg/day experimental groups when compared with control group (p =0.03, p = 0.007, respectively). *Lavandula officinalis* extract significantly decreased serum triglyceride level in the doses of 100, 200 and 400 mg/Kg/day groups when compared with control group (p=0.001, p=0.001, p=0.01, respectively). Serum HDL level was similar in the control and 200, 400 mg/Kg extract groups while higher in 100 mg/Kg group (p=0.01). The serum LDL level was reduced in the 100, 200 and 400 mg/Kg/day extract groups when compared with control group (p=0.001, p=0.001, p=0.007, respectively). *Lavandula officinalis* extract with doses of 100, 200 and 400 mg/Kg/day significantly decreased serum VLDL level when compared with control group (p=0.001, p=0.001, p=0.01, respectively). Lavender extract decreased LDL/HDL level at doses of 100 and 200 mg/Kg/day (p=0.001, p=0.001, respectively). The TG/HDL level decreased in experimental groups with doses of 100 and 200 mg/Kg/day (p=0.001, p=0.000, respectively) (Figures 1 and 2).

## Discussion

In our study lavender extract decreased the serum cholesterol, triglycereide, LDL and VLDL levels in experimental groups. Also Lavender extract improved the LDL/HDL ratio. Lavender extract decreased the LDL/HDL and TG/HDL in experimental groups. Lavender extract increased serum HDL level in dose of 100 mg/Kg/day. It has been identified that high plasma LDL levels are major risk factor for vascular diseases (VD), whereas high levels of HDL are considered a negative risk factor for VD (20). The concentration, the size and the chemical modification of LDL are important for atherogenesis. For more than two decades, it has been known that lipid oxidation plays a central role in atherogenesis. The oxidation hypothesis of atherogenesis has evolved to focus on specific proinflammatory oxidized phospholipids that result from the oxidation of LDL phospholipids containing arachidonic acid. These oxidized phospholipids are largely generated by potent oxidants produced by the lipoxygenase and myeloperoxidase pathways (21). Flavonoids are polyphenolic antioxidants naturally present in vegetables, fruits, and beverages. Flavonoid intake is inversely associated with mortality from coronary heart disease and show an inverse relation with incidence of myocardial infarction (22). Antioxidants such as vitamin E can also reduce free-radical formation by modified LDL (22). Our study indicated that ethanolic extract of* Lavandula officinalis *had high antioxidant property and may reduce free radical formation by decreasing the serum LDL levels. Flavonoids, a class of water-soluble antioxidants, are useful in reducing the risk of coronary heart disease. Flavonoids inhibit oxidation of low-density lipoprotein and reduce thrombotic tendency (22). Total extracts from traditional natural plants, rich in phenolic compounds, have been shown to inhibit the development of atherosclerosis in animal models (23). 

However, data from several studies suggest that water-soluble antioxidants, such as vitamin C, are effective in inhibiting LDL oxidation via the preservation of endogenous antioxidants in LDL (24). Lavender extract may reduce endothelial damage and the severity of atherosclerosis. Oxidative stress via increased production of ROS and LDL oxidation induces inflammatory response (25). Our result showed that Lavender plant had high levels of polyphenols such as phenols, flavonoids and flavonols and high antioxidant capacity that may inhibit LDL oxidation.

The essential oil of lavender plant had potent anti-inflammatory activity against carrageenan in mouse (10). Previous studies showed that lavender oil (100 mg/Kg/day for 5 days) significantly reduced thrombotic events without inducing prohemorrhagic complications at variance with acetylsalicylic acid used as reference drug in mice (26). 

In our study lavender extract decreased the serum cholesterol, triglycereide, LDL and VLDL levels in experimental groups. This is also an effective way to increase the ability to slow down the lipid peroxidation process and to enhance antioxidant enzymes activity (12). Flavonoids, which are mostly hydrophilic and do not bind LDL molecule, either scavenge free radicals or act as chelating agents, or protect Vitamin E, β-carotene and lycopene in the LDL particle or preserve serum paraoxonase activity, that itself promotes hydrolysis of lipid peroxides. Several *in-vitro* studies strongly suggest that phenolics protect LDL from oxidation. Flavonoids, significantly increased LDL lag time and significantly decreased the LDL oxidation rate in healthy adults (27). Evidence linking dietary antioxidants to atherosclerosis in humans is still circumstantial and although in some studies the association of antioxidant intake and low risk for atherosclerosis is perceptible, in others this association cannot be established. 

In the present study, the higher dose of the Lavender extract had lower effect. The exact mechanism is not clear. However, although initial studies suggested that antioxidant supplements might promote health, however, large clinical trials with a limited number of antioxidants detected that excess supplementation with certain putative antioxidants may be harmful (28). Antioxidants in fruits and vegetable, work as a continuous chain to combat the damaging effects of oxidative stress. However, if an antioxidant is not restored by the following antioxidant in the chain, it may become a prooxidant. In this situation, the final effect of such supplementations would be no effect or a damaging effect. Furthermore, sometimes high levels of antioxidants change to prooxidants and act opposite of antioxidants (29,30), which might explain the low effect of higher dose of Lavender in this study.

It should be noted that in the present study, the effect of lavender extract on serum lipids levels in rats with hyperlipidemia was not measured and could be the next target. Also further phytochemical and biological tests are suggested to determine the active chemical constituent responsible for these activities.

## References

[1] Keys A (1997). Coronary heart disease in seven countries. Nutr.

[2] Asgary S, Kelishadi R, Rafieian-Kopaei M, Najafi S, Najafi M, Sahebkar A (2013). Investigation of the lipid-modifying and antiinflammatory effects of Cornus mas L. supplementation on dyslipidemic children and adolescents. Pediatr Cardiol.

[3] Gharipour M, Ramezani MA, Sadeghi M, Khosravi A, Masjedi M, Khosravi-Boroujeni H, Rafieian-Kopaei M, Sarrafzadegan N (2013). Sex based levels of Creactive protein and white blood cell count in subjects with metabolic syndrome: Isfahan Healthy Heart Program. J. Res. Med. Sci.

[4] Setorki, M, Nazari B, Asgary S, Azadbakht L, Rafieian-Kopaei M (2011). Anti atherosclerotic effects of verjuice on hypocholesterolemic rabbits. Afr. J. Pharm. Pharmacol.

[5] Jeffrey L, Anderson, Cynthia D Adams, Elliott M Antman, Charles R Bridges, Donald E Casey, William E Chavey II, Francis M Fesmire, Judith S Hochman, Thomas N Levin, A Michael Lincoff, Eric D Peterson, Pierre Theroux, Nanette Kass Wenger, R Scott Wright (2004). ACC/AHA guidelines for the management of patients with st-elevation myocardial infarction executive summarya report of the american college of cardiology/american heart association task force on practice guidelines (writing committee to revise the 1999 guidelines for the management of patients with acute myocardial infarction). J. Am. Coll. Cardiol.

[6] Madihi Y, Merrikhi A, Baradaran A, Ghobadi S, Shahinfard N, Ansari R, Karimi A Mesripour A, Rafieian-Kopaei M (2013). Bioactive components and the effect of hydroalcoholic extract of Vaccinium myrtillus on postprandial atherosclerosis risk factors in rabbits. Pak. J. Med. Sci.

[7] Nasri H, Shirzad H (2013). Toxicity and safety of medicinal plants. J. Herb. Med. Plarmacol.

[8] Sewell RDE, Rafieian-Kopaei M (2014). The history and ups and downs of herbal medicine usage. J. Herb. Med. Pharmacol.

[9] Omid Baigi R (2000). Production and processing of medicinal plants. Astane Ghods Publicat. Mashhad.

[10] Hajhashemi V, Ghannadi A, Sharif B (2003). Anti-inflammatory and analgesic properties of the leaf extracts and essential oil of Lavandula angustifolia Mill. J. Ethnopharmacol.

[11] Alnamer R, Alaoui K, Bouididael H, Benjouad A, Cherrah Y (2012). Sedative and hypnotic activities of the methanolic and aqueous extracts of lavandula officinalis from morocco. Adv. Pharmacol. Sci.

[12] Wang D, Yuan X, Liu T, Liu L, Hu Y, Wang Z, Zheng Q (2012). Neuroprotective activity of lavender oil on transient focal cerebral ischemia in mice. Molecules.

[13] Asgary S, Rafieian-Kopaei M, Najafi S, Heidarian E, Sahebkar A (2013). Antihyperlipidemic effects of Sesamum indicum L. in rabbits fed a high-fat diet. Sci. World J.

[14] Parsaei P, Karimi M, Asadi SY, Rafieian-Kopaei M (2013). Bioactive components and preventive effect of green tea (Camellia sinensis) extract on postlaparotomy intra-abdominal adhesion in rats. Int. J. Surg.

[15] Sharafati Chaleshtori R, Sharafati Chaleshtori F, Rafieian M (2011). Biological characterization of Iranian walnut (Juglans regia)leaves. Turk. J. Biol.

[16] Asadi SY, Parsaei P, Karimi M, Ezzati S, Zamiri A, Mohammadizadeh F, Rafieian-Kopaei M (2013). Effect of green tea (Camellia sinensis) extract on healing process of surgical wounds in rat. Int. J. Surg.

[17] Akhlaghi M, Shabanian G, Rafieian-kopaei M, Parvin N, Saadat M, Akhlaghi M (2011). Citrus aurantium blossom and preoperative anxiety. Rev. Bras. Anestesiol.

[18] Heidarian E, Rafieian-Kopaei M, Ashrafi K (2013). The Effect of hydroalcoholic extract of allium latifolium on the liver phosphatidate phosphatase and serum lipid profile in hyperlipidemic rats. J. Babol. Univ. Med. Sci.

[19] Asgary S, Rafieian-Kopaei M, Shamsi F, Najafi S, Sahebkar A (2014). Biochemical and histopathological study of the anti-hyperglycemic and anti-hyperlipidemic effects of cornelian cherry (Cornus mas L.) in alloxan-induced diabetic rats. J. Compl. Integr. Med.

[20] Navab M, Ananthramaiah GM, Reddy ST, Van Lenten BJ, Ansell BJ, Fonarow GC, Vahabzadeh K, Hama S, Hough G, Kamranpour N (2004). Thematic review series: the pathogenesis of atherosclerosis the oxidation hypothesis of atherogenesis: the role of oxidized phospholipids and HDL. J. Lipid Res.

[21] Hertog MG, Kromhout D, Aravanis C, Blackburn H, Buzina R, Fidanza F, Giampaoli S, Jansen A, Menotti A, Nedeljkovic S (1995). Flavonoid intake and long-term risk of coronary heart disease and cancer in the seven countries study. Arch. Int. Med.

[22] Nasri H, Sahinfard N, Rafieian M, Rafieian S, Shirzad M, Rafieian-kopaei M (2013). Effects of Allium sativum on liver enzymes and atherosclerotic risk factors. J. Herb. Med. Pharmacol.

[23] Nasri H, Rafieian-Kopaei M (2013). Oxidative stress and aging prevention. Int. J. Prev. Med.

[24] Asgary S, Sahebkar A, Afshani M, Keshvari M, Haghjooyjavanmard Sh, Mahmoud Rafieian-Kopaei M (2013). Clinical evaluation of blood pressure lowering, endothelial function improving, hypolipidemic and anti-inflammatory effects of pomegranate juice in hypertensive subjects. Phytother. Res.

[25] Ballabeni V, Tognolini M, Chiavarini M, Impicciatore M, Bruni R, Bianchi A, Barocelli E (2004). Novel antiplatelet and antithrombotic activities of essential oil from Lavandula hybrida Reverchon. Phytomed.

[26] Andrikopoulos NK, Kaliora AC, Assimopoulou AN, Papageorgiou VP (2002). Inhibitory activity of minor polyphenolic and nonpolyphenolic constituents of olive oil against in-vitro low-density lipoprotein oxidation. J. Med. Food.

[27] Rafieian-Kopaei M, Baradaran A, Rafieian M (2013). Oxidative stress and the paradoxical effects of antioxidants. J. Res. Med. Sci.

[28] Rafieian-Kopaie M, Baradaran A (2013). Plants antioxidants: From laboratory to clinic. J. Nephropathol.

[29] Azadmehr A, Ziaee A, Ghanei L, Fallah Huseini H, Hajiaghaee R, Tavakoli-far B, Kordafshari GH (2014). A Randomized clinical trial study: anti-oxidant, anti-hyperglycemic and anti hyperlipidemic effects of olibanum Gum in type 2 diabetic patients. Iran. J. Pharm. Res.

[30] Mohadjerani M (2012). Antioxidant activity and total phenolic Content of Nerium oleander L. Grown in North of Iran. Iran. J. Pharm. Res.

